# Removal of Bursæ of the Wrist by the Operation of Extirpation

**Published:** 1885-07

**Authors:** Charles C. F. Gay


### Clinical Reports.

### REMOVAL OF BURSÆ OF THE WRIST BY THE OPERATION OF EXTIRPATION.

BY CHARLES C. F. GAY, M. D.

At a recent meeting of the New York Surgical Society, *Dr. A. C. Post narrated the case of bursal swelling at the back of the wrist, for which he operated successfully by puncturing the cyst, whereupon Dr. Sands "inquired how frequently the members of the society had performed cutting operations for the cure of this disease, as it seemed to be a question of considerable interest at the present time; that his knowledge of extirpation of such ganglia had been limited to two cases; the first case was successful, but the second case left the hand nearly useless."

*Feb. 20, 1885, *New York Medical Journal*, March 14th, page 312.

The question propounded by Dr. Sands—accompanied by the statement that a hand had been rendered useless after the operation of extirpation—has induced me to look through my record of cases of operations by compression, free or subcutaneous incision or puncture, ligature and extirpation. I have selected two representative cases of the latter, and transcribe them from my notes precisely as they were recorded at the time, or immediately after the operations were made, although subcutaneous incision may be included in the term "cutting operation," yet the doctor has reference only to operation by extirpation; hence, other methods of cutting, of which I have notes, will not be here considered.

### Case I.

*Bursa upon the Front of the Right Wrist, of Four Years' Duration.—Operation by Extirpation.*

Mr. B., *act.* thirty years, has tumor upon the front of the wrist, which began to show itself four years ago. The tumor is now large and extends out upon the palm of the hand. There is numbness of two fingers, and when the fingers are extended there is much pain; for this reason, the fingers are kept flexed. Three years ago, the patient was treated three times by application of electrolysis, which was very painful and served no good purpose. At my office I etherized the patient, applied Esmarch's bandage, and, assisted by Dr. Bartow, made an incision over the tumor one and a half inches in length. Dissection was carried forward, care being taken not to open the sheath of the tendon until it was found impracticable to remove the sac entire, when the sac was opened and its contents evacuated, which consisted of a teaspoonful of matter resembling boiled rice. The larger portion of the sac was now excised, leaving a small part of it in the wound. The edges of the wound were brought together and secured by two silver sutures, and dressed by wet compress and bandage. Three months after the operation, the wrist was entirely well and strong, and has remained so ever since, with no recurrence of the disease; it being now about six years since the operation was made.

### Case II.

*Bursa of the Back of the Wrist, of Two Years' Duration.—Operation by Extirpation.*

Miss A. G., *act.* 30 years, has a bursa upon the back of the wrist, about the size of a large hickory nut, located chiefly upon the ulner border of the wrist, which has become very troublesome and painful. She thinks it was caused by lifting a tub of water. At my office, the patient was etherized, Esmarch's bandage applied, when, assisted by Dr. Warren, I removed the greater portion of the sac by careful dissection.

Believing the function of the joint would be imperiled by attempt to remove the sac entire, it was excised, leaving a small portion of it in the wound. The sac contained a thick, translucent fluid. The wound was closed and secured by two silver sutures, and dressed by wet compress and bandage. Three months afterwards, the cure was perfect. There has been no return of the disease, three and a half years since the operation; the wrist being quite as strong as it was before the operation.

At the time I performed these operations, I thought I had strong ground for suspecting a recurrence of the disease, inasmuch as I was obliged to leave a portion of the sac behind, which might serve as a nucleus for another growth, but the results show that my suspicions were unfounded. The successful issue of these cases may help to resolve doubts as to the propriety of the operation by extirpation. If there be no danger of recurrence of the disease, when a portion of the sac is allowed to remain, it is the best operation for the cure of bursal tumors of the wrist. The age of the tumor will, however, influence the surgeon in making choice of the method of removing the tumor. The contents of a recent cyst will be more fluid and less concrete in character than the contents of an old cyst; hence, subcutaneous punction may be advisable for the former, and extirpation in case of the latter.

